# Mutation at G103 of MtbFtsZ Altered their Sensitivity to Coumarins

**DOI:** 10.3389/fmicb.2017.00578

**Published:** 2017-04-06

**Authors:** Duggirala Sridevi, Karpagam U. Sudhakar, Ragamanvitha Ananthathatmula, Rakesh P. Nankar, Mukesh Doble

**Affiliations:** Bioengineering and Drug Design Lab, Department of Biotechnology, Indian Institute of Technology MadrasChennai, India

**Keywords:** MtbFtsZ, G103, site-directed mutagenesis, MIC, IC_50_, protofilament formation

## Abstract

Coumarins are natural polyphenol lactones comprising of fused rings of benzene and α-pyrone. The current study demonstrates the inhibitory effect of coumarins with various substitutions on *Mycobacterium smegmatis* mc^2^ 155. We also demonstrate the effect of pomegranate (*Punica granatum*) extract containing ellagic acid, on *M. smegmatis* as well as their affect on MtbFtsZ (FtsZ from *Mycobacterium tuberculosis*). The ellagic acid extracts from pomegranate peels inhibit mycobacteria with a MIC of 25 μM and 0.3 to 3.5 mg/mL, respectively, but failed to inhibit the polymerization of MtbFtsZ. However, the coumarins were shown to inhibit the polymerization and GTPase activity of the protein as well as have an inhibitory affect on *M. smegmatis* mc^2^ 155. Docking of the most active coumarin (7-Dimethyl-4-methyl coumarin with MIC of 38.7 μM) to the GTP binding site suggests that it interacted with the G103 residue. Based on the docking results two mutants of varying activity (G103S and G103A) were constructed to elucidate the interaction of MtbFtsZ and coumarins. Mutation of G103 with Serine (a bulky group) results in an inactive mutant and substitution with alanine produces a variant that retains most of the activity of the wild type. There is a disruption of the protofilament formation of the MtbFtsZ upon interaction with coumarins as demonstrated by TEM. The coumarins increase the length of Mycobacteria five times and MtbFtsZ localization is disturbed. The mutant proteins altered the GTPase and polymerization activity of coumarins as compared to wild type protein. The results here support that coumarins inhibit proliferation of Mycobacteria by targeting the assembly of MtbFtsZ and provide the possible binding site of coumarins on MtbFtsZ. This study may aid in the design of natural products as anti-mycobacterial agents. The currently reported GTP analogs for FtsZ are toxic to the human cell lines; natural coumarins targeting the GTP binding site of MtbFtsZ may hold promise as an important drug lead for tuberculosis treatment.

## Introduction

Tuberculosis is the second leading infectious disease with highest mortality in the world, caused by *Mycobacterium tuberculosis* (Zumla, 2015). The emergence of X/MDR strains (Extensively/multi drug-resistant TB) has complicated the scenario thereby necessitating several groups to focus on newer targets, synthesis of novel compounds and explore natural products that can serve as leads for the design of anti-tubercular drugs (Palomino et al., 2009; Lima et al., 2015; Zumla, 2015). Among the targets, Filamentous temperature-sensitive mutant Z (FtsZ) protein is an essential bacterial protein which comprises of nucleotide binding N and C-terminal domains with the (tubulin)-like synergy loop (T7 loop). FtsZ is involved in Z-ring formation, which is formed after the segregation of nuclear material during the membrane constriction (Egan et al., 2013). The amino acid motif, GGGTGTG is similar to the signature sequence of tubulin—thus termed as bacterial tubulin homolog (Egan et al., 2013; Hong and Xie, 2013). Its nucleotide binding region is involved in assembly of the individual monomers to form a protofilament. FtsZ is a self-activating GTPase and the catalytic site is formed by the interaction of two monomers in a head to tail manner, where in the GTP binding domain interacts with the T7 loop of the adjacent monomer (Bernander and Ettema, 2010). The protofilament thus formed will interact to form bundle, pairs, sheets that is the integral part of the “Z”-ring (Chan et al., 2014). Inhibition of FtsZ leads to cell elongation and eventually death of the organism thus motivating many research groups to focus on the design of inhibitors targeting this enzyme (Leung et al., 2000, 2004; Jaiswal et al., 2007; Chan et al., 2015). Cinnamaldehyde, berberine, curcumin, viriditoxin, and chrysophaentins A-H, are reported to be potent inhibitors against a wide range of bacteria and they target the GTPase and the polymerization ability of FtsZ (Artola et al., 2015; Li and Ma, 2015). Structure based virtual screening of small molecule libraries identified 3-alkoxylbenzamide derivatives as potent inhibitors of MRSA strains of *Staphylococcus*, and PC190723 is the most promising compound inhibiting the FtsZ GTPase activity (Anderson et al., 2012; Stokes et al., 2013; Singh et al., 2014). Drug discovery studies that focussed on scaffolds such as naphthalenes, benzimidazoles, and pyrimidopyrazines targeting FtsZ were reasonably successful (Jaiswal et al., 2007). Quinoloines and taxanes were reported to specifically inhibit MtbFtsZ (Sun et al., 2014). Screening of known tubulin inhibitors against *M. tuberculosis* identified derivatives of pyridopyrazine and pteridine. They inhibit Mycobacteria by targeting the MtbFtsZ protein (Mathew et al., 2011). Recently we reported the role of dihydroquinolines in inhibiting Mycobacteria by targeting MtbFtsZ (Duggirala et al., 2016).

Coumarins are a set of natural compounds found in different plants with as many as 1300 of them being identified as secondary metabolites (Keri et al., 2015). Chemically they are synthesized by Wittig's and Reformatorsky's reactions (Lv et al., 2014). Coumarins find varied applications as additives in foods, perfumes, cosmetics, dyes and pharmaceuticals. The unique versatile scaffold of coumarin backbone is prone to modifications and derivatization with several functional groups, thereby leading to construction of structure based libraries (Min et al., 2012). They can bind to many ligands and are categorized as “privileged structures” thus affecting many biological functions such as inflammatory process, redox balance, blood coagulation, apoptosis etc (Peng et al., 2013; Vazquez-Rodriguez et al., 2015). Their pharmacological properties are attributed to the 2*H*-chromen-2-one nucleus composed of the lactone and aromatic ring. The two oxygen atoms in the lactone ring interact with the important amino acid residues of enzymes thereby impacting the biological processes. Drugs in the market possessing the coumarin scaffold are anticoagulants namely acenocoumarol, dicoumarol, warfarin and antibiotic such as novobiocin (Gellert et al., 1976). The aromatic ring forms hydrophobic interaction with the carbon residues of the protein to destabilize the enzyme (Musa et al., 2008; Riveiro et al., 2010). Coumarins with long chain hydrocarbon substitution such as ostruthin, ammoresinol, anthogenol, novobiocin, coumermycin, and chartreusin demonstrate anti-bacterial activity on many gram positive and gram negative bacteria (Matos et al., 2012; Garro et al., 2014). Although none of the commercially available anti-TB drugs possess coumarin scaffold, scopoletin and umbelliferone are active against *Mycobacterium tuberculosis* H_37_Rv, with MIC values of 42 and 58.3 μg/mL, respectively (Rezayan et al., 2012; Upadhyay et al., 2012). The biological activity of coumarins and their target depends on substitution patterns of the functional groups (Venugopala et al., 2013). Target based drug therapy is well explored in drug discovery programs, hence the characterization of coumarins as potent anti-mycobacterial agents and elucidating their target, would add strength to the search for novel drugs.

Pomogranate was known for its anti-bacterial, anti-viral, anti-cancer, anti-inflammatory, anti-oxidant properties. Pomegranate peel is a rich source of tannins, flavonoids and polyphenols (Ambigaipalan et al., 2016). Ethanolic extract and methanol extract of pomegranate husk are a good source of ellagic acid (Nankar and Doble, 2015). The ethanol extracts from the peels possess anti-bacterial activity against the salmonella strains (Wafa et al., 2017). *In lieu* of the anti-bacterial properties of the pomegranate peels, here we use the ethanol and methanol extracts to analyse their anti-mycobacterial property. Earlier we reported the anti-bacterial effect of natural coumarins and identified their role in targeting the FtsZ from *E. coli* (Duggirala et al., 2014). Here we screened these compounds and pure ellagic acid from pomegranate extracts (*Punica granatum*) for their inhibitory effect on Mycobacteria and MtbFtsZ. The coumarins inhibited the activity of MtbFtsZ and the mutations altered their sensitivity further. Based on studies on the mutant enzymes we propose that the binding site of coumarins at MtbFtsZ could be G103 residue.

## Materials and methods

GTP and IPTG were purchased from Sigma, St. Louis, USA. Middlebrook 7H9 Broth and 7H10 agar media base were purchased from BD, Biosciences USA. Coumarins were purchased from TCI chemicals, Japan. DMSO was purchased from Merck, USA. All other chemicals are of analytical grade. Different coumarins with substitutions at the 7^th^ position were chosen as indicated previously (Duggirala et al., 2014).

### Expression and purification of MtbFtsZ

pSAR 1 plasmid harboring MtbFtsZ gene in pET15 vector is a kind gift from Dr. Malini Rajagoplan, University of Texas, USA. Protein was expressed in E. coli BL21 (DE3) Rosetta cells. MtbFtsZ is a soluble protein and it was expressed by induction of IPTG, and purified by Ni-NTA column chromatography (Chen et al., 2007; Duggirala et al., 2016). The protein was analyzed on SDS-PAGE and its purity was determined to be >95%. The gel was visualized in BioRad gel dock system and Quantity one 4.6.9 software. Protein concentration was measured by BCA method and stored at −80°C until further use The protein was stable in 25 mM HEPES buffer (pH 6.5), 50 mM KCl and 5 mM MgCl2. (Rajagopalan et al., 2005).

### Preparation of pomegranate peels extract

Peels of kesar pomegranate fruit were obtained from Andavar fruit shop, IIT Madras Campus, Chennai, India. Methanol extract (MethExt) and ethyl acetate fraction of methanol extract (EthAcFr) were prepared from pomegranate peels as mentioned previously (Nankar and Doble, 2015). MethExt and EthAcFr contain 2% w/w and 17% w/w of ellagic acid, respectively. The extracts were lyophilized and the powder was suspended in DMSO. These two extracts and pure ellagic acid were screened for their ability to inhibit Mycobacteria.

### Docking of coumarins

The crystal structure of MtbFtsZ (PDB ID- 2Q1Y) was chosen as the target for docking of coumarins. The protein was downloaded and prepared for docking by removing the water molecules and subsequently minimizing its energy in Molecular operating environment (MOE), 2014.08; Chemical Computing Group, Canada). The structures of coumarins were sketched in Chem Draw (Chem Draw, Chambridge, Soft, USA) and the energy was minimized using MMF94X force field available in MOE. The GTP binding site was defined by forming a cavity around it and the bound GTP was removed and the coumarins were docked at this site. For docking in the T7 loop a 10 A° radius was chosen around the major T7 loop residue. Docking was performed using the program Genetic Optimization for Ligand Docking (GOLD), suite 5.2 (CCDC, UK) (Gupta et al., 2011). The best GOLD scores for each of the inhibitors were reported here.

### Site directed mutagenesis of G103 residue of MtbFtsZ

Quick change site directed mutagenesis kit (Agilent, technologies) was used to mutate the MtbFtsZ gene at G103 position. pSAR1 plasmid was used as the template to induce mutation at the G103 site to generate G103S and G103A mutants. The primers for the mutations are listed in Table 1.

**Table 1:** **Primers used in the study**.

**Construct**	**Primers**
G103S	FP - GTCACCGCCGGCGAG AGC GGCGGAACCGGCACC RP - GGTGCCGGTTCCGCC GCT CTCGCCGGCGGTGAC
G103A	FP - GTCACCGCCGGCGAG GCG GGCGGAACCGGCACC RP - GGTGCCGGTTCCGCC CGC CTCGCCGGCGGTGAC

The PCR products were subjected to *dpn1* digestion and the resultant products were transformed in *E. coli* DH5α cells. The plasmid was isolated from the resultant colonies and the mutation was confirmed by full-length plasmid sequencing (Eurofins, India). The protein was expressed in the same host as wild type, purified and concentrated as per the protocol used for the wild type (Chen et al., 2007).

### Effect of coumarins on the polymerization activity of MtbFtsZ

The ability of the coumarins to inhibit the polymerization and GTPase activity of MtbFtsZ was measured (both wild type and mutant proteins). 6 μM of MtbFtsZ, was polymerized in 25 mM HEPES buffer, pH 6.5, 50 mM KCl and 5 mM MgCl2 on ice, in the presence and absence of compounds. The polymerization reaction was monitored in a fluorescence spectrophotometer (JASCO 6500) with both the excitation and emission wavelengths set at 400 nm, and temperature set at 30°C. At the end of 300 s 1 mM of GTP was added and the light scattering was recorded till 1,000 s (Jaiswal et al., 2007).

### Effect of coumarins on the GTPase activity of MtbFtsZ

6 μM of MtbFtsZ (wild type and mutant proteins) was incubated in MOPS buffer (50 mM MOPS pH 7.2, 5 mM MgCl2, 200 mM KCl) in the presence and absence of coumarins. GTP was added and the reaction was incubated at 37°C for 15 min. BIOMOL Malachite green reagent (BIOMOL® Green reagent, Enzo Life Sciences, USA) was used to measure the free inorganic phosphate released (Matsui et al., 2012). The absorbance at 620 nm was measured after the addition of 100 μl of BIOMOL reagent. A standard curve was prepared with pure KH2PO4.

### Determination of IC_50_

The concentration of coumarins required to inhibit both the polymerization and GTPase activities of MtbFtsZ by 50% (IC50) was estimated. The coumarins were diluted from 10 to 200 μM while maintaining the DMSO concentration at 1%. The IC50 for each of the compound was estimated by fitting the data obtained between concentrations vs. activity using GraphPad Prisim- version 5. The values represented here is the mean of three independent experiments. The Km and Vmax were also calculated in GraphPad Prisim.

### Inhibitory effect of coumarins on mycobacteria

*Mycobacterium smegmatis* mc^2^ 155 (ATCC 14468) was obtained from IMTECH, Chandigarh, India. The susceptibility of this strain toward coumarins was analyzed by Resazurin based Microtiter alamar blue assay (MABA). The bacteria were maintained in Middlebrook 7H9 medium (Difco Laboratories, Detroit, MI, USA), supplemented with OADC (oleic acid/bovine albumin, dextrose, catalase). Rifampicin and streptomycin were included as positive control. Coumarins were diluted in either DMSO or sterile deionized water. The concentration of DMSO was maintained at 1%. One hundred microliter of 7H9 broth was dispensed in each well of a sterile flat bottom 96-well plate and a serial 2-fold dilution of each of these coumarins was added directly in the wells. The cells of *M. smegmatis* were adjusted to late logarithmic phase (A600 > 0.6) and used for the assay. Untreated cells served as positive control. The plates were incubated at 37°C for 48 h. Alamar blue (10% of the total culture volume) was added to each well. A change in color from blue to pink indicated the growth of bacteria and A_570_ was measured in Enspire Multimode plate reader (PerkinElmer, NJ, USA). MIC was determined based on the lowest concentration of coumarin where no color change was observed. The effect of coumarins on the bacterial cell length was analyzed by incubating the late logarithmic *M. smegmatis* cells with 50 μM of coumarins and the cells were fixed and mounted to be viewed under a 100 X objective of Carl-Zeiss Bright field Microscope (Axio Imager 2 for Life Science Research, Germany).

### Transmission electron microscopy

The polymerization of MtbFtsZ was visualized with TEM. 5 μM of MtbFtsZ in HEPES buffer, in the presence/absence of 100 μM of esculetin and 7-dimethyl-4-methyl coumarin was incubated with GTP (1 mM) for 10 min and applied to a carbon-coated copper grid (300 mesh). They were stained with freshly prepared 2% of sodium tungstate. The grids were blotted dry and the specimens were viewed with Philips JE, 301 electron microscope at × 50,000 magnification.

### Effect of coumarins on the FtsZ localization of *M. smegmatis* mc^2^ 155

The effect of coumarins on the Z-ring formation of *M. smegmatis* was determined with immunofluoresence microscopy using anti-FtsZ antibodies (Dziedzic et al., 2010). Late logarithmic phase cells were grown in the presence and absence of coumarins for 4 h. The cells were fixed with formaldehyde washed with phosphate buffer, and immuno-stained with polyclonal anti-FtsZ rabbit antibody (GeneTeX, USA) overnight at 4°C. Alexa fluor (488) goat anti-rabbit secondary antibody (Invitrogen, USA) was used for visualizing Z-rings. The nuclear material was visualized by using an anti-mountant containing DAPI. The cells were visualized in 63 X objective using a Carl-Zeiss fluorescence Microscope (Axio Imager 2 for Life Science Research, Germany). The images were visualized and processed using ZEN image processing software.

## Results

### Site-directed mutagenesis at the GTP binding site

MtbFtsZ was mutated at the G103 residue by site directed mutagenesis based on literature report that mutations at this residue alter the GTPase activity of the enzyme (Lu et al., 2001). In addition to the inactive mutant (G103S) another mutant with an amino acid alanine (G103A) substitution which may not completely affect the activity of the protein was also constructed. The plasmid sequencing was performed to confirm the mutation at G103 residue.

### Docking score of coumarins with MtbFtsZ

**Figure 1:**
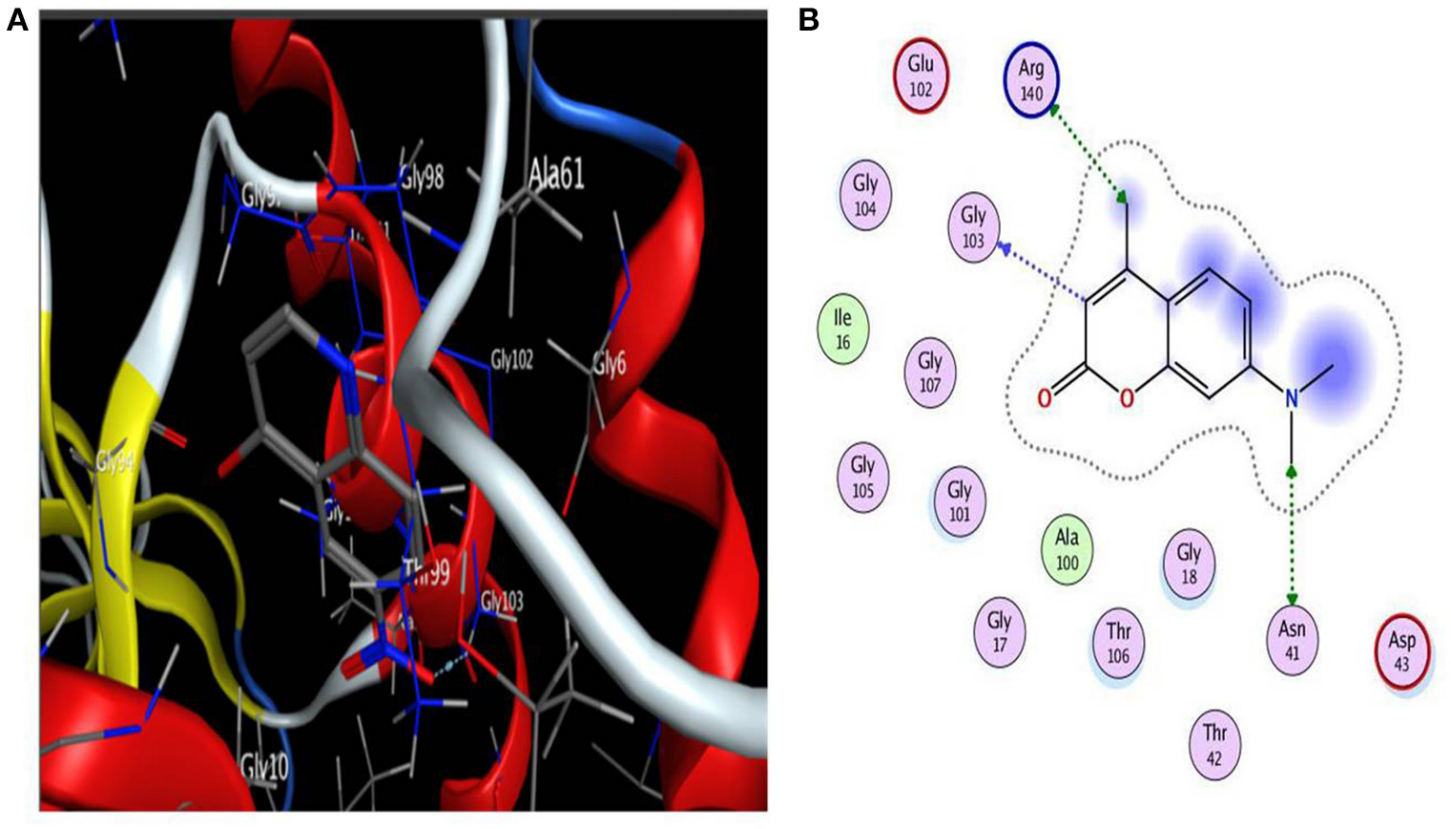
**(A)** Interaction of 7-diethyl-4-methylcoumarin with GTP binding site of MtbFtsZ. **(B)** Indicates the Ligplot representing the interaction of 7-diethyl-4-methylcoumarin with the G103 residue of the GTP binding site. Interaction is through hydrogen bonding. Docking was done using GOLD software.

Based on literature docking of the coumarins was performed at the putative GTP binding site of MtbFtsZ (PDB ID 2Q1Y) using the GOLD (Genetic Optimization for Ligand Docking) software (CCDC). The GOLD score predicts the binding energy values of the ligands with the protein. Among all the coumarins 7-Dimethyl-4-methylcoumarin and Daphnetin bind to MtbFtsZ with the highest docking score (around 42) and they interact with the G103 residue through hydrogen bonding (Figure 1). The coumarins such as 4-Hydroxycoumarin and coumarin interact poorly with a lower GOLD score value (Table 2). The results are represented as GOLD score fitness which is an original scoring function in GOLD. It predicts the ligand binding positions by taking into account the H-bonding energy, van der Waals energy, metal interaction and ligand torsion strain.

**Table 2:** **Coumarins docked to the GTP binding site of MtbFtsZ (2Q1Y) using GOLD software, [a], The GOLD score is given as average of 5 independent runs**.

**Compound**	**GOLD score^a^**
Esculetin	37.5
Umbelliferone	35.5
4-Methylumbelliferone	32.9
4-Hydroxycoumarin	31.8
Coumarin	31.8
7-Diethylamino-4- methylcoumarin	40.1
6-Methylcoumarin	39.7
Daphnetin	42.4
7-Dimethyl-4-methylcoumarin	42.8

### Functional characterization of mutants

**Figure 2:**
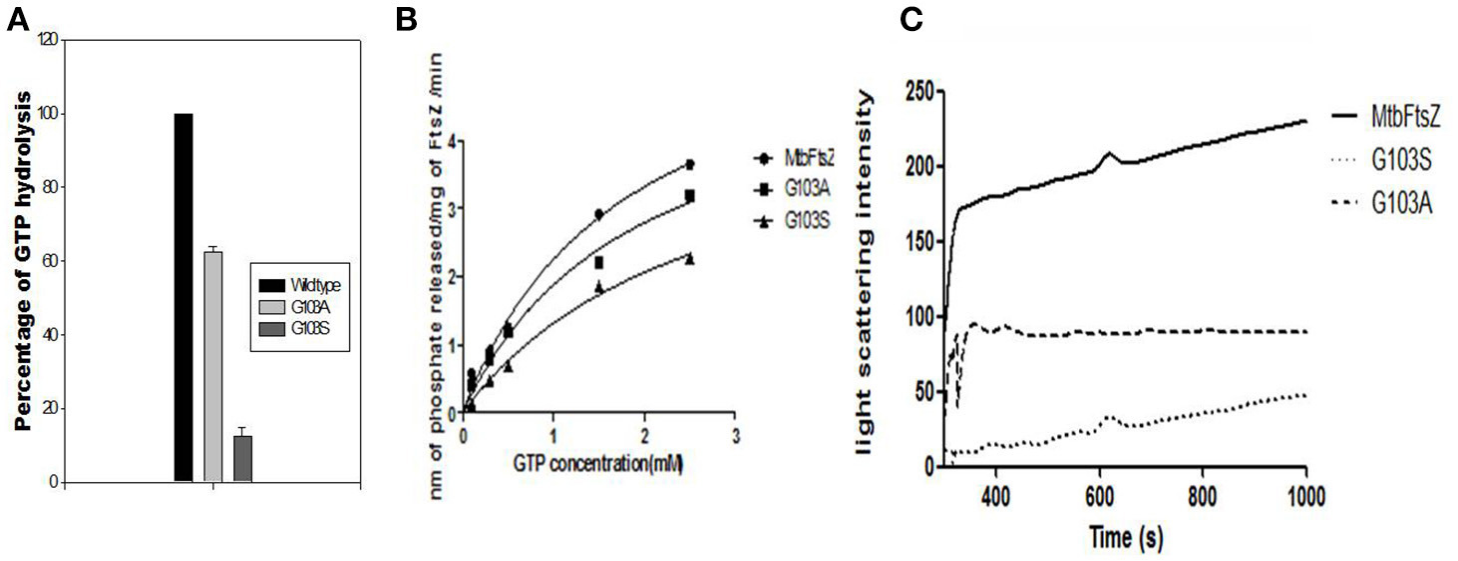
**Functional characterization of G103S and G103A mutants of MtbFtsZ. (A)** GTPase activity of the mutant proteins is expressed as percentage with respect to wild protein. **(B)** Represents the kinetics of the mutant and the wild type proteins as analyzed by GTPase hydrolysis assay. **(C)** Represents the polymerization activity. Light scattering intensity is represented here which is a measure of the polymerization activity of the protein. The polymerization activity represented here is the light intensity from 400 to 1,000 s. The graph in **(A,B)** is representative of three independent experiments.

G103 residue was demonstrated to play a role in MtbFtsZ (Rajagopalan et al., 2005). Thus, we chose to mutate MtbFtsZ at this site and generate two mutants of varying activity. The activity of the mutants was analyzed by GTP hydrolysis assay and the light scattering assay. The G103S mutant and G103A retained 10% and 65% of the original (wild type) GTPase activity respectively (Figure 2A). MtbFtsZ (wild type) yielded 3.64 nM of inorganic phosphate/min/mg of protein, with a Km of 1.78 ± 0.46 nM and a V_max_ of 6.28 ± 0.8 nM/min. The Km for G103S and G103A are 2.6 ± 0.72 nM and 1.8 ± 0.56 nM respectively while the corresponding V_max_ is 4.7 ± 0.78 and 5.4 ± 0.86 nM/minute respectively (Figure 2B). The two mutants retained 8.3% and 40% of the wild type polymerization activity respectively. The intensity of light scattering is proportional to the polymerization of the protein. Wild type MtbFtsZ polymerized the maximum with the highest light scattering intensity while G103A polymerized to a lesser extent, and the G103S polymerized the least (Figure 2C).

### Effect of coumarins on the polymerization and GTPase activity of MtbFtsZ mutants

The IC_50_ values for the polymerization activity of MtbFtsZ in the presence of different coumarins with substitutions at 7^th^ position range from 60 to 125 μM. 7-Dimethyl-4-methylcoumarin exhibited the maximum inhibition of the polymerization activity of the protein in a dose dependent manner with an IC_50_ value of 62.5 ± 6.7 μM followed by esculetin (65 ± 4.6 μM). The effect of coumarins on the GTPase activity is similar with IC_50_ ranging from 60 to 135 μM. Daphnetin, esculetin and 7-dimethyl-4-methylcoumarin inhibited the GTPase activity of the protein the best (Table 3). Berberine and Totarol, two reported inhibitors of FtsZ were used as positive controls for both the assays. The former inhibited 75% ± 2.5 and 82% ± 1.4 of the GTPase and polymerization activity of the protein respectively, whereas the corresponding activity of the latter is 68% ± 3.1 and 74% ± 2 respectively (Jaiswal et al., 2007; Stokes et al., 2013).

**Table 3:** **Effect of coumarins on GTPase and polymerization activity of MtbFtsZ**.

**Compound**	polymerization activity^*^ IC_50_ ± SD (μM)			GTPaseactivity^**^ IC_50_ ± SD (μM)		
	**Wild type**	**G103S**	**G103A**	**Wild type**	**G103S**	**G103A**
Esculetin	84.6 ± 4.7	48.5 ± 2.5	74 ± 5.3	65 ± 4.6	55.5 ± 2.4	69.8 ± 1.7
Umbelliferone	124.2 ± 7.5	52.8 ± 3.8	85 ± 4.72.7	135.2 ± 5.9	52.8 ± 3.5	85 ± 2.3
4-Methylumbelliferone	99.2 ± 3.2	102.4 ± 2.7	112.6 ± 3.4	125.1 ± 4.9	102.4 ± 6.7	112.6 ± 3.9
4-Hydroxycoumarin	>200	>200	150 ± 6.4	198 ± 3.5	>200	178.7
Coumarin	>200	73 ± 6.6	89 ± 5.4	>200	69.5 ± 5.7	88.3 ± 2.9
7-Diethylamino-4-methyl coumarin	81.54 ± 6.4	65.2 ± 1.8	72.5 ± 3.9	110.3 ± 0.3	78.47 ± 1.8	57.97 ± 3.5
6-Methylcoumarin	>200	124.7 ± 6.7	148.4 ± 3.6	>200	136.1 ± 2.9	178.4 ± 2.1
Daphnetin	89.5 ± 6.3	55.2 ± 5.4	78.7 ± 6.1	62.5 ± 5.8	89.5 ± 5.2	94.7 ± 2.9
7-Dimethyl-4-methyl coumarin	74.1 ± 2.8	64.1 ± 2.9	60.9 ± 4.8	64.47 ± 6.7	45.7 ± 1.8	44.2 ± 5.4

The IC_50_ values were calculated for the polymerization activity and GTPase activity by light scattering assay^*^ and Malachite green assay^**^ respectively as indicated in experimental section. Data here is represented as mean ± SD of three independent experiments.

Table 3 indicates the effect of coumarins on the wild type and the two mutant proteins. The IC_50_ was estimated for all coumarins using both the assays. There is a direct correlation between the inhibition of GTPase and the polymerization activities of MtbFtsZ by coumarins. The coumarins lacking a substitution at the 7^th^ position however did not inhibit the activity of the enzyme. Similar results were obtained with the FtsZ from *E. coli*. The coumarins which did not alter the enzyme activity of wild type protein such as the 4-hydroxycoumarin had no effect on the activity of the G103A and G103S mutants (IC_50_ > 200 μM) as well. But some of them such as Daphnetin, Esculetin, and Umbelliferone exhibit variation in IC_50_ values among the wild type and mutant proteins.

### TEM analysis of the MtbFtsZ

**Figure 3:**
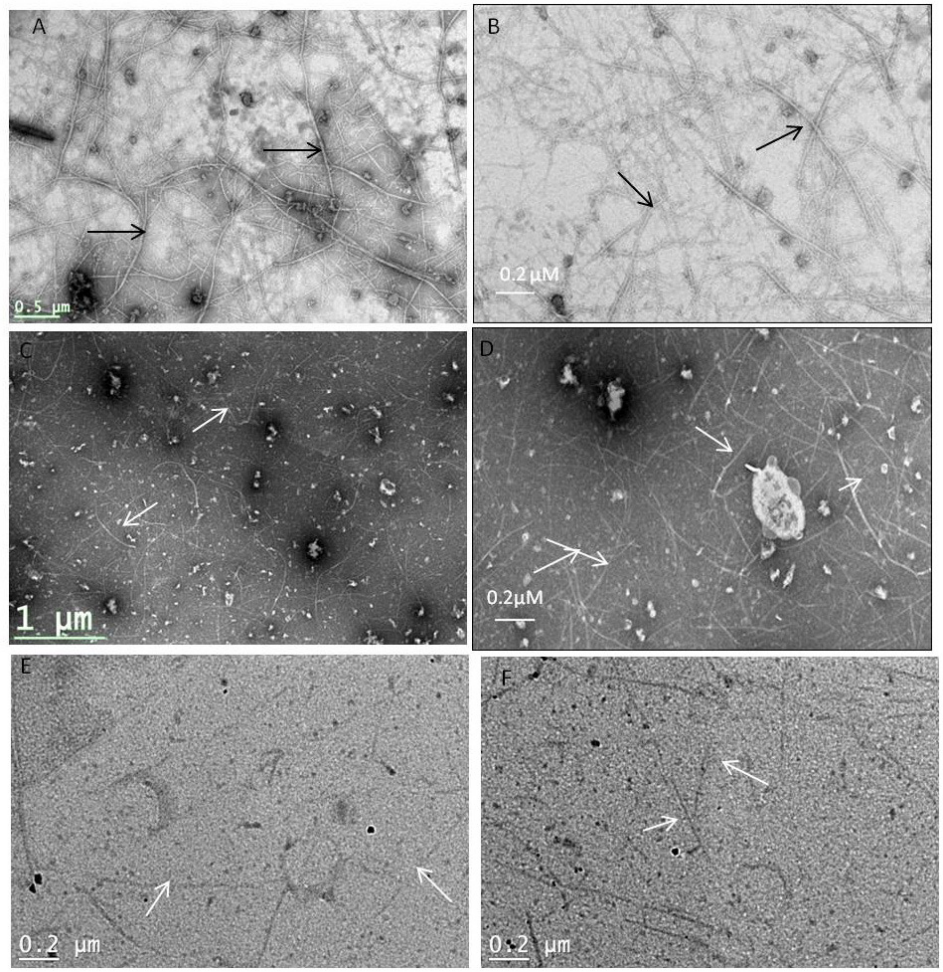
**Protofilament formation of MtbFtsZwild type and mitant proteins**. MtbFtsZ wild type and mutant proteins were polymerized in the presence of 1 mM GTP in HMK buffer in the absence and presence of coumarins. **(A,B)** Indicates the protofilament formation of wild typeMtbFtsZ (wild type protein where the monomers are thick and form a profuse network). **(C,D)** Represent the polymerization of MtbFtsZ in the presence of esculetin and 7-dimethyl-4-methylcoumarin where the monomers are thin and the network is absent. **(E,F)** Represent the G103A and G103S proteins. Scale bar for **(B,D)** 0.2 μM. The arrows point to the polymerized filaments of protein in case of wildtype and unpolymerized monomers in case of mutants.

TEM images confirm that coumarins strongly inhibit the polymerization and assembly of MtbFtsZ protofilament (Figure 3). The FtsZ monomers of wild type protein are thick and appear as a profuse bundle to form a protofilament in the absence of inhibitor (Figures 3A,B). The presence of esculetin and 7-dimethyl-4-methylcoumarin did not allow the formation of network for a protofilament. The filaments are thin and scattered (Figures 3C,D). Natural products such as sanguinarine and totarol are reported to induce similar effect on FtsZ protofilament formation (Jaiswal et al., 2007). G103A mutant polymerizes to a lesser extent than the wild type to form a network with thin filaments (Figure 3E). There is no polymerization observed in the case of G103S mutant and the monomers are thin (Figure 3F).

**Figure 4:**
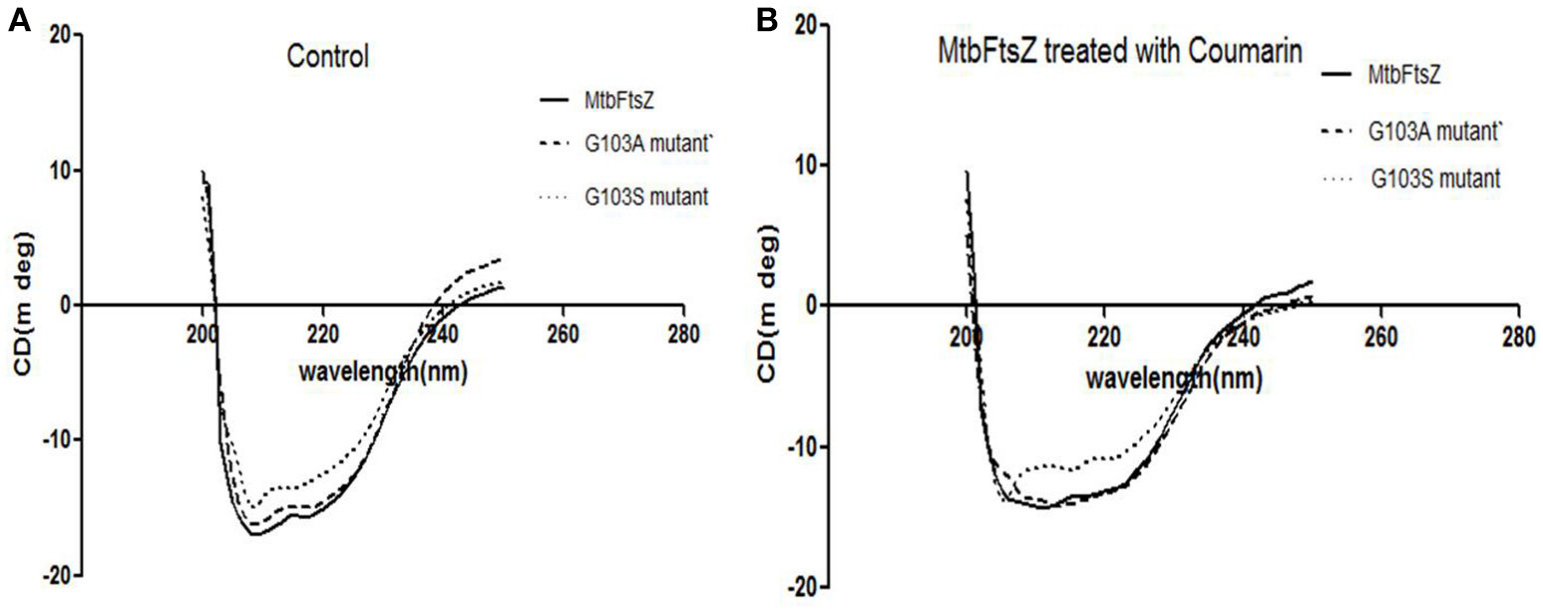
**CD-spectroscopy for the mutant and wild type MtbFtsZ proteins. (A)** Indicates the CD spectrum in the absence of coumarin. **(B)** Indicates the CD spectrum of wild type and mutant proteins upon treatment with coumarin, 7-dimethyl-4methyl coumarin.

The secondary structure of the protein was altered upon mutation at the G103 residue with serine as indicated previously (Figure 4A). The troughs at 208 and 220 nm are characteristic of the alpha helix of the protein, thus a dip at these regions is an indication of alteration of the secondary structure (Figure 4B). The characteristic high alpha helix was disturbed upon mutation and the dip was more apparent by addition of the 7-dimethyl-4-methyl coumarin.

### Coumarins inhibit growth and alter the morphology of *Mycobacterium smegmatis* mc^2^ 155

The effect of coumarins, ellagic acid, Methanol extract (MethExt) and ethyl acetate fraction (EthAcFr) of pomegranate peels on the proliferation of *Mycobacterium smegmatis* mc^2^ 155 was studied (Table 4). The basic coumarin and 4-hydroxycoumarin exhibit very poor activity with very high MIC (Table 4). However, substitutions, especially at the 7^th^ position, have an inhibitory effect on this bacterium. 7-Dimethyl-4-methyl coumarin is the most active in this series with a MIC of 36.8 μM (Table 4). Recent reports have indicated that the crude extracts and fractions of *Terminalia* species, from South Africa inhibit *M. smegmatis* growth (Fyhrquist et al., 2014). The dominant component among them is ellagic acid in addition to tannins such as punicalagin, punicalin, chebulanin, chebulagic acid, chebulinic acid, corilagin, terflavin, terchebulin, and tellimagrandin. All these compounds were reported to possess anti-microbial activity. Hence in the current study pure ellagic acid along with pomegranate extract (containing ellagic extract) were screened for their ability to inhibit the growth of *M. smegmatis* mc^2^ 155. Pure ellagic acid inhibits *M. smegmatis* mc^2^ 155 with a MIC of 50 μM. However, The MethExt and EthAcFr extract from pomegranate peel inhibit with a MIC of 3.35 mg/mL and 0.395 mg/mL. These extracts possess 2 and 17% of ellagic acid, respectively. EthAcFr has lower MIC value when compared to the reported MIC (1.5 mg/mL) of butanol extract from the bark of the *Terminalia* species. The tannins present in the crude extracts probably augment the inhibitory effect of ellagic acid (Fyhrquist et al., 2014).

**Table 4:** **Inhibitory effect of coumarins and pomegranate extracts on ***M. smegmatis*** mc^**2**^**.

**Compounds**	**MIC (μM)**
Esculetin	48.2 ± 5.7
Umbelliferone	46.2 ± 3.2
4-Methylumbelliferone	42.5 ± 2.4
4-Hydroxylcoumarin	150.2 ± 7.4
Coumarin	213.8 ± 8.7
7-Diethylamino-4-methylcoumarin	70.2 ± 6.2
6-Methylcoumarin	46.8 ± 4.3
Daphnetin	48.2 ± 6.3
7-Dimethyl-4-methylcoumarin	36.8 ± 7.1
Ellagic acid	50 ± 2.4
EthAcFr	0.395 mg/mL
MethEx	3.35 mg/mL

*Rifampicin (MIC – 3.9 μM) and streptomycin (MIC – 11.17 μM) were used as positive control. Methanol extract (MethExt) and ethyl acetate fraction (EthAcFr) refers to the ellagic acid from pomegranate peels as mentioned in Section Materials and Methods*.

**Figure 5:**
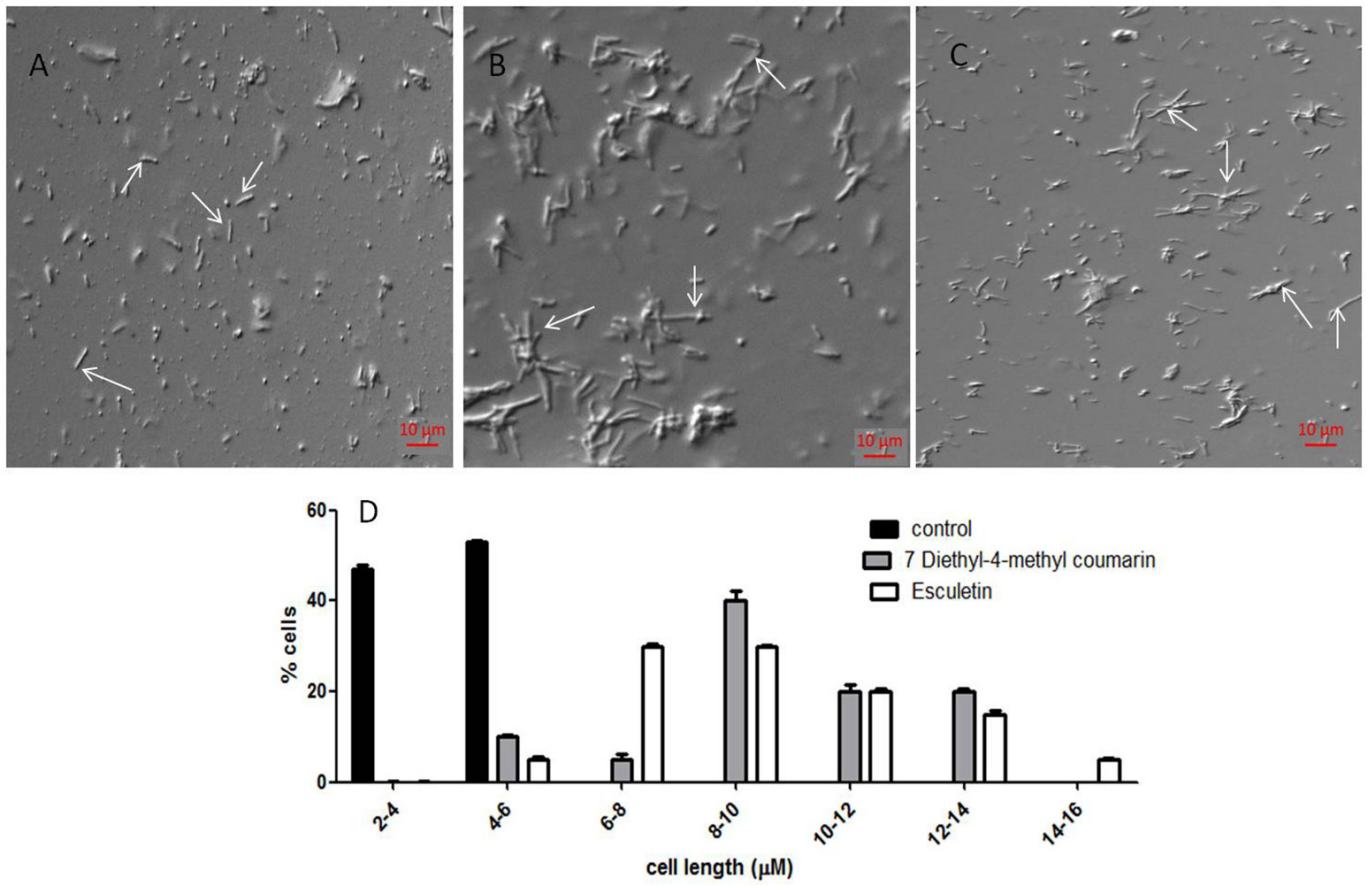
**Effect of coumarins on the morphology of ***M. smegmatis*** mc^**2**^ 155—Mid-logarithmic phase cells of ***Mycobacterium smegmatis*** were treated with esculetin and 7-dimethyl-4-methylcoumarin. (A)** Control (treated with DMSO), **(B,C)** represent cells treated with 50 μM of esculetin and 7-dimethyl-4-methylcoumarin respectively. **(D)** Represents the histograms of distribution of cell length of the control and treated bacteria as measured by ImageJ. Three different fields were observed for each of the panel and 100 bacterial cells were counted per field.

Coumarins also alter the length of *M. smegmatis* mc^2^ 155. The average length of the cells without treatment is 4.48 ± 1.25 μm (Figure 5A), However upon treatment with esculetin and 7-dimethyl-4-methylcoumarin the maximum bacterial length increased 4-fold (17.85 ± 4.5 μm and 15.2 ± 3.5 μm respectively) (Figures 5B,C). The length of the cells was measured in ImageJ software. The distribution of cell length is represented in a histogram where the control cells are maximum in number at the lower cell length range (2–4 μM) and are absent as the cell length increases. The cell length distribution of control and coumarin treated cells indicate that coumarins could induce filamentation in *M. smegmatis* mc^2^ 155. The length of *Bacillus subtilis* 168 was also reported to increase when treated with coumarins (Duggirala et al., 2014; Figure 5D).

### Localization of MtbFtsZ in *Mycobacterium smegmatis*

**Figure 6:**
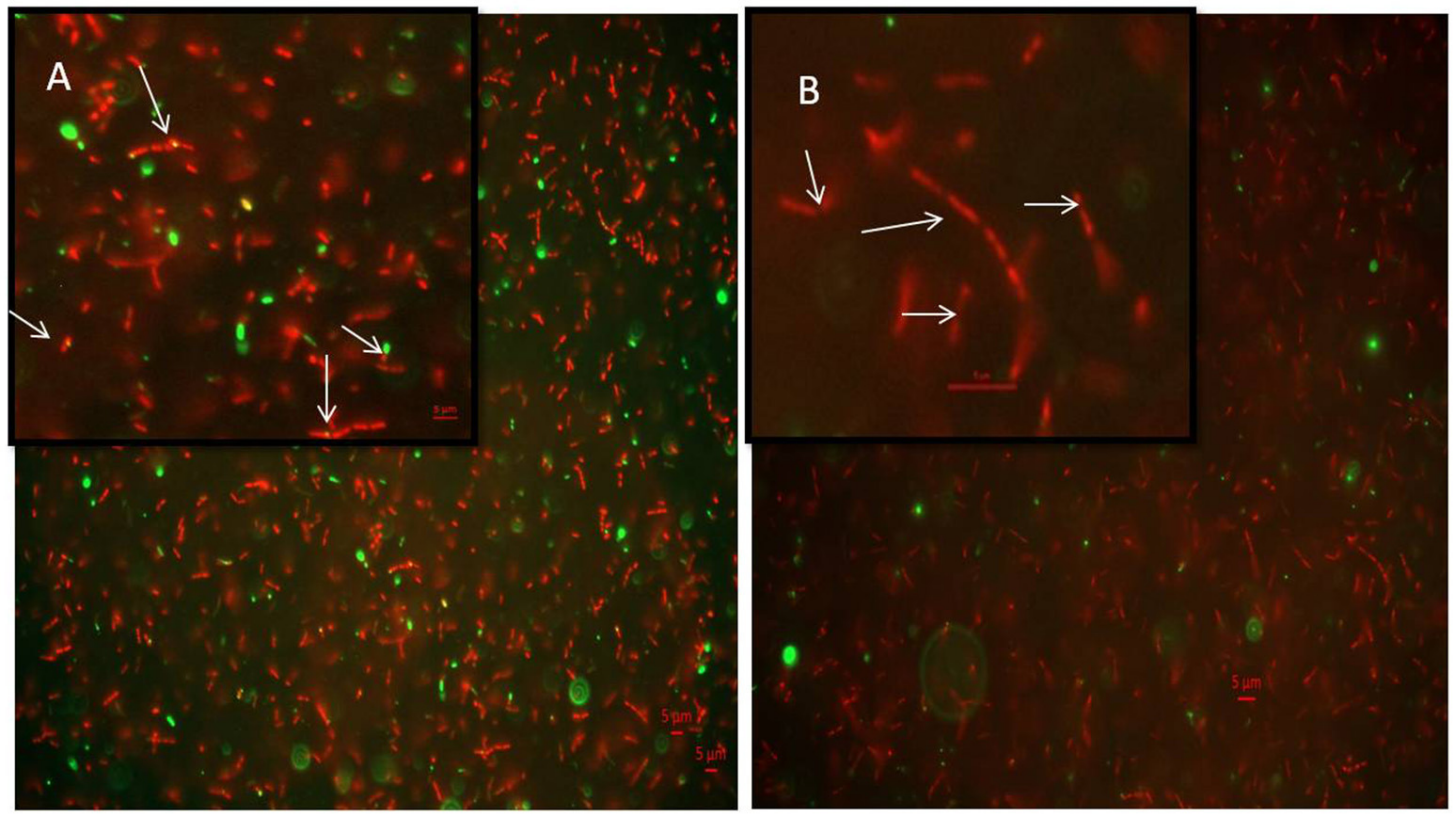
**Immunolocalization of MtbFtsZ in ***M. smegmatis*** (A)** untreated and **(B)** treated with 7-dimethyl-4-methyl coumarin. The above figures are the merged images of the dyes. FtsZ is seen as a green spots all over the cells as seen in the inset and they are not observed in the treated cells. Inset represents the single bacteria of interest where MtbFtsZ is seen clearly in the control cells **(A)** Scale 5 μM. Images were obtained in Carl – Zeiss microscope fitted with AxioCam 506. The filters used were 493–520 nm for alexa fluor and 358–463 nm for DAPI. The arrows indicate the localization of FtsZ in the control cells which is absent in cells treated with coumarin.

7-dimethyl-4-methylcoumarin inhibits the growth of *M. smegmatis* by altering the length of the *Mycobacterial* cells as well as disrupting the activity of MtbFtsZ. Hence its effect on FtsZ assembly in growing cultures of *M. smegmatis* was analyzed by immunofluoresence microscopy as indicated in methodology. FtsZ is stained as a bright green spot in the center of the cells in the untreated cells (Figure 6A). In the cells treated with this 7-dimethyl-4-methylcoumarin, FtsZ is distributed thought out the cell as seen as bright green spots (Figure 6B). Thus, the localization of MtbFtsZ was disrupted when compared with the control cells.

## Discussion

With the increase in drug resistance there is a need for novel drugs and those from natural compounds are the need of hour as they are non-toxic to the host system. Target based screening of small molecules is an important tool in drug discovery programmes. Many studies focused on screening natural compounds, and synthesized small molecules from combinatorial libraries. FtsZ is one of the essential proteins during cell division. It serves as a scaffold during the formation of divisome. There is a growing amount of literature characterizing FtsZ. It has unique virtues to serve as a target. It is conserved in all bacterial species; hence an inhibitor could serve as broad spectrum antibiotic. It shares less sequence homology with the eukaryotic homolog tubulin thus it can specifically target bacterial species. Reduced functional activity of FtsZ results in inhibition of cell division and cells with filamentous phonotype are observed (Araújo-Bazán et al., 2016).

Many drug discovery labs are focusing on identification of anti-TB drugs and many drugs targeting specific enzymes and pathways are being added to the pipeline. One promising target for tuberculosis treatment is MtbFtsZ (Mathew et al., 2016). Though many compounds were reported as FtsZ inhibitors not many are on their way to clinical trials. Through a systematic approach we characterize coumarins as anti-mycobacterial compounds and elucidate their mechanism of action on MtbFtsZ. The compounds interacted with G103 residue of the GTP binding site as demonstrated by Ligplot (Figure 1B). Site directed mutagenesis was performed at G103 residue to generate variant proteins with single amino acid substitution namely the G103A and G103S mutants. The polymerization activity and the GTPase activity of the MtbFtsZ wild type and the mutant proteins in the presence of coumarins were analyzed. The extracted coumarins and the selected coumarins which are reported to have good biological activity were analyzed for their ability to inhibit the proliferation of *Mycobacterium smegmatis* mc^2^ 155.

The assembly of FtsZ is GTP driven process. GTP inhibitors such as C8 analogs employed as FtsZ inhibitors, do not interfere with tubulin assembly (Marcelo et al., 2013). However, they are less active on the bacteria as they could not enter them due to high hydrophobicity of the cell wall. Artola et al. (2015) synthesized compounds with naphthalene group that replace GTP as well as inhibit *B. subtilis* suggesting that they can penetrate the membrane (Artola et al., 2015). Our docking analysis indicates that coumarins can bind with good affinity at the GTP binding site and glycine plays a distinct functional role on the protein by interacting with the phosphate. Substitution of glycine with serine during site directed mutagenesis will destabilize the activity of the enzyme since the latter is a bulky moiety. Whereas, substitution of glycine with alanine, is reported to partly retain the activity of enzyme. Moreover, the secondary structure of the protein remains unaltered upon substituting with alanine (Ganter and Plückthun, 1990). Site directed mutagenesis was well employed to study the role of vital residues in FtsZ. For ex, mutagenesis at single Cys-155 of MtbFtsZ to alanine reduced the assembly of *Mtb*FtsZ (Jaiswal and Panda, 2008). Mutations at the GTP binding site and T7 loop domain of MtbFtsZ altered the activity of the protein both *in vivo* and *in vitro*. G103S mutant was reported to reduce the GTPase activity of the protein as compared to the wild type (Rajagopalan et al., 2005). It was reported that G103S protein which is defective in GTP hydrolysis does not form Z ring assembly *in vivo*. Highlighting the importance of this residue we chose to generate two variant mutants from the same residue, and we analyzed the activity of the coumarins on the mutant proteins and wild type.

The enzyme kinetics studies showed that Km of G103A is closer to that of the wild type indicating that affinity of the protein to GTP is retained by this mutant, whereas the Km of G103S is high with a lower V_max_ indicating that the affinity of GTP to this mutant has decreased (decreasing its activity by 40%). And the activity of G103A mutated protein was decreased only by 22%. Glycine is known to bind with the phosphate of the GTP to enable its hydrolysis and substitution with serine reduces the binding ability of GTP with the protein (Rajagopalan et al., 2005) whereas substitution with alanine does not affect the binding ability of GTP to the protein, thus reducing the activity of the enzyme only partly. It is observed that the kinetics of assembly of MtbFtsZ is much slower than that of EcFtsZ, namely, the former hydrolyses GTP at a much slower rate than the latter (White et al., 2000). Polymerization activity is measured by the light scattering intensity and the peaks of same are achieved for EcFtsZ within 50 s while it took 900 s for the MtbFtsZ (Duggirala et al., 2014). The assembly kinetics of MtbFtsZ is independent of calcium, and the turnover of GTP is much slower with it when compared to that of EcFtsZ (Jaiswal and Panda, 2009). The coumarins lacking the functional group at the 7^th^ position did not inhibit the activity of MtbFtsZ and EcFtsZ.

The coumarins had the maximum effect on the wild type enzyme. The mutation at the G103 residue altered the IC_50_ values of the coumarins resulting in low drug concentration requirement for inhibition when compared to wild type. Thus, the sensitivity of the enzyme to the coumarins was altered upon mutating at the G103 residue hence emphasizing the importance of glycine residue on protein activity as well its interaction with coumarins.

The TEM images are in support of GTPase and polymerization activities where 7-dimethyl-4-methyl coumarin exhibits a decrease in IC_50_ for the mutants (Table 3). Plumbagin and totarol are reported to induce bactericidal effect by increasing the length of bacteria by 7-fold and 5-fold respectively (Jaiswal et al., 2007; Bhattacharya et al., 2013). A similar observation is represented here with esculetin and 7-dimethyl-4-methylcoumarin indicating a 4-fold increase of *M. smegmatis* mc^2^ 155 length. These compounds are reported to primarily target the FtsZ and induce bacterial cell death. Thus, coumarins also seem to induce cell elongation and inhibit mycobacterial growth by perturbing the assembly of MtbFtsZ thereby inducing cell death. FtsZ inhibitors such as Taxanes, zantrins and viriditoxins also possess anti-bacterial activity with MIC ranging from 0.25 to 10 μM toward *Bacillus subtilis* or pathogenic bacteria such as *S. aureus* (Huang et al., 2006; Li and Ma, 2015). The immunofluoresence studies of FtsZ protein tagged with anti-FtsZ antibodies indicates that 7-dimethyl-4-methyl coumarin blocked FtsZ assembly and protofilament formation thus inhibiting cell division as FtsZ is seen distributed throughout the cells (seen as green spots in Figure 6B). These microscopic studies collectively show the inhibitory activity of 7-dimethyl-4-methylcoumarin on MtbFtsZ protein in support of the IC_50_ values of these compounds on MtbFtsZ.

## Conclusion

Tuberculosis is the most deadly infectious disease and the emergence of MDR-strains due to irrational usage of drugs has complicated the scenario. With the urgent need to discover new drugs with improved efficacy as well as novel specific targets, FtsZ has received much attention as a promising novel drug target perhaps due to the low sequence homology with tubulin. FtsZ in presence of GTP interact with rest of the proteins viz; FtsA, SulA and ZAP proteins to form the constriction of membrane. Even though such analogs are absent in MtbFtsZ, the mutation of the key residue altered the sensitivity of the coumarins. The current study indicates that coumarins can be well explored as leads for tuberculosis therapy. They are also known to be non-toxic to mammalian cells. Ellagic acid extracts from Pomegranate peels affect the *Mycobacterium* more effectively but the tannins could have precipitated the protein. Chrysophaentins which are non-nucleotide competitive inhibitors binding to the GTP binding site have been reported. Two variants of MtbFtsZ (G103S and G103A) were constructed and the effects of coumarins on the polymerization and GTPase activity of these were studied to understand the mechanism. The coumarins perturbed the assembly of MtbFtsZ and the mutation further destabilzed the secondary structure and polymerization of protein.

## Author contributions

DS initiated the idea and performed the site directed mutagenesis of key residues of MtbFtsZ. DS and MD designed the study. RN isolated and characterized the ellagic acid from the pomogranate peels. DS and KS performed MABA assays. KS estimated the IC_50_ scores for the wild type protein and RA estimated the score for mutant proteins and she performed the CD experiments.

## Funding

The above work is supported by DST-WOSA grant—SR/WOS-A/LS-188/2012.

### Conflict of interest statement

The authors declare that the research was conducted in the absence of any commercial or financial relationships that could be construed as a potential conflict of interest.
